# Leukocytoclastic Vasculitis in a Patient with Type 1 Cryoglobulinemia

**DOI:** 10.1155/2011/124940

**Published:** 2011-10-10

**Authors:** Paul Y. Liu, Pamela E. Prete, Gary Kukes

**Affiliations:** ^1^Division of Rheumatology, Department of Medicine, University of California, Irvine, Orange, CA 92868, USA; ^2^Veterans Administration Healthcare System, Long Beach, CA 90822, USA

## Abstract

Cutaneous manifestations of type 1 cryoglobulinemia are usually related to vascular occlusion by noninflammatory thrombosis; rarely is leukocytoclastic vasculitis seen in type 1 cryoglobulinemia. We report the case of a 64-year-old male who presented with isolated cutaneous leukocytoclastic vasculitis that was initially attributed to essential mixed cryoglobulinemia after thorough diagnostic evaluation. A lack of adequate clinical response to therapy prompted further investigation, including cryoprecipitate electrophoresis and immunofixation, which revealed an IgM kappa monoclonal gammopathy consistent with type 1 cryoglobulinemia. A renewed search for an underlying malignancy led to the discovery of early Waldenstrom's macroglobulinemia. Although leukocytoclastic vasculitis is more characteristic of mixed cryoglobulinemia, it can be a presenting manifestation of type 1 cryoglobulinemia.

## 1. Introduction

Cutaneous manifestations of type 1 cryoglobulinemia are usually related vascular occlusion and include Raynaud's phenomenon, acrocyanosis, cutaneous infarctions, and necrotic ulcerations [1]. Histologically, noninflammatory thrombosis is typically seen in type 1 cryoglobulinemia whereas leukocytoclastic vasculitis is common in mixed cryoglobulinemia [1]. We report a patient with type 1 cryoglobulinemia secondary to Waldenstrom's macroglobulinemia that presented with cutaneous leukocytoclastic vasculitis.

## 2. Case Report

A 64-year-old male presented with fatigue, bilateral leg swelling, and a painful lower extremity rash for one month. Examination revealed 1 to 5 millimeter macules that were tender, violaceous, and nonblanching. The patient had no history of recent infections, new medications, or other systemic complaints. Treatment of the rash with topical steroids was not successful.

**Figure 1:**
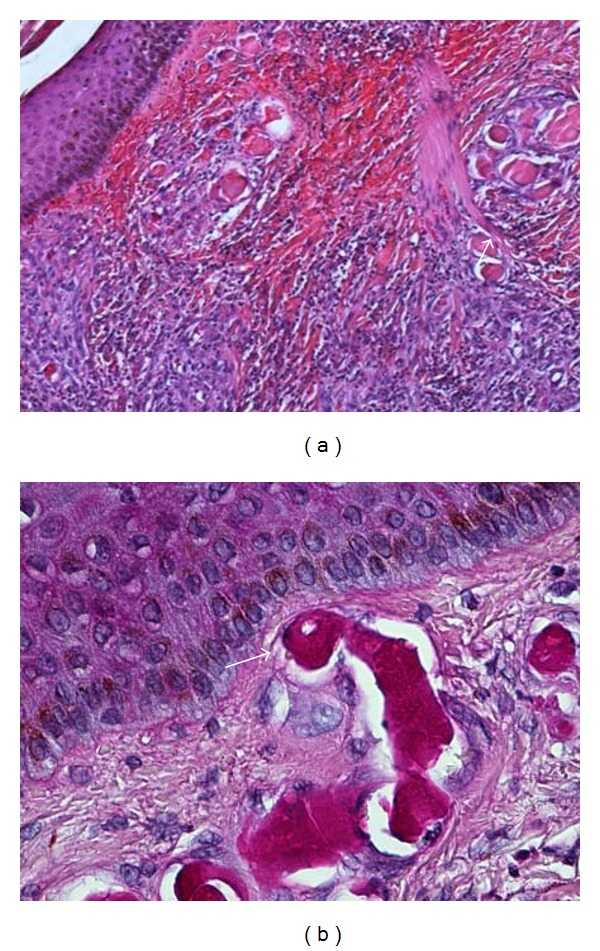
(a) Leukocytoclastic vasculitis of thin walled, papillary dermal vessels characterized by mural fibrinoid necrosis and nuclear dust (arrow). A marked perivascular neutrophilic infiltrate and many extravasated red blood cells are present (hematoxylin and eosin stain; 100×). (b) Intraluminal monoclonal immunoglobulin casts in superficial papillary dermal vessels. Note swollen endothelial cells (arrow) (periodic acid-schiff stain, 400×).

Complete blood count, creatinine, liver enzymes, urinalysis, prothrombin time, partial thromboplastin time, thyroid function tests, and lactate dehydrogenase levels were all within normal limits. Hepatitis B and C serologies and human immunodeficiency virus tests were negative. Antinuclear antibodies and subsets, rheumatoid factor, and antineutrophilic cytoplasmic antibodies were negative. Erythrocyte sedimentation rate was 47 mm/hr, C-reactive protein was 1.8 mg/dL, beta-2 microglobulin was 3.1 mg/L (0–3 mg/L), C3 was 139 mg/dL, and C4 was 18.8 mg/dL (20–47 mg/dL). Serum cryoglobulins were present at 14%. Quantitative serum immunoglobulin testing showed an elevated IgM of 1770 mg/dL, and serum viscosity was normal at 1.56. A skin punch biopsy revealed leukocytoclastic vasculitis with cryoglobulin deposits (Figure 1). Computed tomography of the chest, abdomen, and pelvis was normal as was a bone marrow aspirate.

Treatment for presumed essential mixed cryoglobulinemia with oral prednisone 40 mg and azathioprine 150 mg daily resulted in only a partial response, and attempts at tapering steroids were unsuccessful. The lack of response after two months of therapy prompted further testing including a serum protein electrophoresis which revealed an IgM kappa monoclonal gammopathy (0.7 gm/dL). Electrophoresis of the cryoglobulin precipitate was then performed which revealed an IgM kappa monoclonal gammopathy (5.98 gm/dL) consistent with type 1 cryoglobulinemia. At our request, a repeat bone marrow aspiration was performed which demonstrated a 1% abnormal population of monoclonal B cells consistent with early Waldenstrom's macroglobulinemia. Treatment with four weekly doses of rituximab 500 mg IV resulted in a gradual resolution of the rash coupled with a decreased IgM kappa to 0.2 gm/dL within three months, and prednisone was subsequently tapered off.

After six months of remission, macules reappeared on the lower extremities but did not respond to a second course rituximab and steroids. Other unsuccessful therapies included methotrexate, two cycles of bortezomib 1.3 mg/m^2^ IV, and a combination of melphalan, thalidomide, and prednisone. After being lost to follow up for one year, he was admitted with lower extremity ulcerations secondary to worsening cutaneous vasculitis and received solumedrol 80 mg IV daily plus plasmapheresis. Quantitative cryoglobulins decreased from 17.5% to 2%, and the patient improved clinically. Anticoagulation was also initiated for a right lower extremity deep venous thrombosis. He was readmitted eight months later for infected cutaneous ulcers and severe sepsis that necessitated intravenous antibiotics and surgical debridement. As a result of sepsis, he developed new-onset renal failure requiring chronic dialysis. Presently, the patient continues to receive periodic plasmapheresis for cryoglobulinemic vasculitis.

## 3. Discussion

Type I cryoglobulins, which account for approximately 20% of cryoglobulinemia cases, are monoclonal immunoglobulins that reversibly precipitate at temperatures below 37°C and are typically associated with an underlying lymphoproliferative disorder such as Waldenstrom's macroglobulinemia, multiple myeloma, or chronic lymphocytic leukemia [3]. Cutaneous manifestations are common and consist of macules, papules, infarctions, hemorrhagic crusts, and ulcerations [3, 4]. Histopathology in type 1 cryoglobulinemia is usually characterized by the presence of non-inflammatory hyaline thrombosis and cryoglobulin deposits whereas leukocytoclastic vasculitis is characteristic of mixed cryoglobulinemia [1, 4, 5]. Our patient presented with lower extremity macules that progressed to cutaneous ulcers. His skin biopsy revealed cryoglobulin deposits which can be seen in type 1 cryoglobulinemia. Interestingly, leukocytoclastic vasculitis was also appreciated, which is typically not observed in type 1 cryoglobulinemia.  Table 1 summarizes the differential diagnosis of cutaneous vasculitis as well as that of vascular occlusive disease. It is important to note that the presence of histologic cryoglobulin deposits and the lack of response to steroids prompted further testing that led to the diagnosis of type 1 cryoglobulinemia and, ultimately, Waldenstrom's macroglobulinemia.

Treatment of cryoglobulinemia should be focused on the underlying disease process if possible. Cutaneous involvement may be an indication for immunosuppressive therapy. A number of pharmacologic agents have been used including systemic steroids and cyclophosphamide [3]. Rituximab, a chimeric antibody that targets CD20 antigen on B cells, has also been shown to be effective in treating mixed cryoglobulinemia [6, 7]. However, its efficacy in type I cryoglobulinemia is less clear [8, 9]. Plasmapheresis is indicated for severe or life-threatening complications of cryoprecipitation. It has also been used as an effective adjunctive therapy in recalcitrant type I cryoglobulinemia [3]. Though our patient responded to an initial trial of rituximab, repeat therapy for recurring disease was ineffective and plasmapheresis was required to achieve an adequate clinical response.

## Figures and Tables

**Table 1:** Diseases associated with cutaneous leukocytoclastic vasculitis.

Vasculitic drug Reactions	Miscellaneous
Examples: propylthiouracil, methimazole, antimicrobials	Urticarial vasculitis
	Hematologic malignancies
Systemic vasculitides	Inflammatory bowel disease
Mixed cryoglobulinemia	
Wegener's granulomatosis	Diseases associated with cutaneous vascular occlusion
Microscopic polyangiitis	
Churg-Strauss syndrome	Thrombotic
Henoch-Schönlein purpura	Type 1 cryoglobulinemia
Polyarteritis nodosa	Antiphospholipid antibody syndrome
Behcet's disease	Sickle cell disease
	Thrombotic thrombocytopenic purpura
Connective Tissue Diseases	Warfarin-induced skin necrosis
Systemic Lupus Erythematosus	Livedo vasculopathy
Rheumatoid Arthritis	Disseminated intravascular coagulation
Sjögren's Syndrome	
Relapsing Polychondritis	Embolic
	Cholesterol embolus
Infections	Atrial myxoma
Hepatitis B/C	
Human immunodeficiency virus	Vessel wall pathology
Cytomegalovirus	Calciphylaxis
Ebstein-Barr Virus	Amyloidosis
Parvovirus B-19	Radiation arteriopathy
Endocarditis	Primary hyperoxaluria

Adapted from Carlson [2].

## References

[1] Pol-Rodriguez MM, Crane S, Feinberg DL, Glusac EJ, Bolognia JL (2003). Retiform purpura. *Archives of Dermatology*.

[2] Carlson JA (2010). The histological assessment of cutaneous vasculitis. *Histopathology*.

[3] Vila AT, Barnadas MA, Ballarin J (2004). Cutaneous ulcers with type I cryoglobulinemia treated with plasmapheresis. *European Journal of Dermatology*.

[4] Cohen SJ, Pittelkow MR, Su WPD (1991). Cutaneous manifestations of cryoglobulinemia: clinical and histopathologic study of seventy-two patients. *Journal of the American Academy of Dermatology*.

[5] Resnik KS (2009). Intravascular eosinophilic deposits-when common knowledge is insufficient to render a diagnosis. *American Journal of Dermatopathology*.

[6] De Vita S, Quartuccio L, Fabris M (2007). Rituximab in mixed cryoglobulinemia: increased experience and perspectives. *Digestive and Liver Disease*.

[7] Zaja F, De Vita S, Mazzaro C (2003). Efficacy and safety of rituximab in type II mixed cryoglobulinemia. *Blood*.

[8] Rosenthal E, Pesce A, Karsenti JM, Allieri-Rosenthal MA, Cassuto JP (2001). Polyneuropathy and vasculitis associated with IgG type I cryoglobulinemia can be treated effectively withthe anti-CD20 monoclonal antibody (rituximab). *Blood*.

[9] Nehme-Schuster H, Korganow AS, Pasquali JL, Martin T (2005). Rituximab inefficiency during type I cryoglobulinaemia. *Rheumatology*.

